# Improving fine-grained food classification using deep residual learning and selective state space models

**DOI:** 10.1371/journal.pone.0322695

**Published:** 2025-05-05

**Authors:** Chi-Sheng Chen, Guan-Ying Chen, Dong Zhou, Di Jiang, Daishi Chen, Shao-Hsuan Chang

**Affiliations:** 1 Neuro Industry, Inc., San Francisco, California, United States of America; 2 Graduate Institute of Biomedical Electronics and Bioinformatics, National Taiwan University, Taipei, Taiwan; 3 School of Engineering, University of Liverpool, Liverpool, United Kingdom; 4 Department of Otolaryngology, Dongguan People’s Hospital, Dongguan, Guangdong, China; 5 Department of Otolaryngology, Shenzhen People’s Hospital, Shenzhen, Guangdong, China; 6 Professional Master’s Program of Biotechnology Management, National Taiwan University, Taipei, Taiwan; Jiangsu Open University, CHINA

## Abstract

**Background:**

Food classification is the foundation for developing food vision tasks and plays a key role in the burgeoning field of computational nutrition. Due to the complexity of food requiring fine-grained classification, the Convolutional Neural Networks (CNNs) backbone needs additional structural design, whereas Vision Transformers (ViTs), containing the self-attention module, has increased computational complexity.

**Methods:**

We propose a ResVMamba model and validate its performance on processing complex food dataset. Unlike previous fine-grained classification models that heavily rely on attention mechanisms or hierarchical feature extraction, our method leverages a novel residual learning strategy within a state-space framework to improve representation learning. This approach enables the model to efficiently capture both global and local dependencies, surpassing the computational efficiency of Vision Transformers (ViTs) while maintaining high accuracy. We introduce an academically underestimated food dataset CNFOOD-241, and compare the CNFOOD-241 with other food databases.

**Results:**

The proposed ResVMamba surpasses current state-of-the-art (SOTA) models, achieving a Top-1 classification accuracy of 81.70% and a Top-5 accuracy of 96.83%. Our findings elucidate that our proposed methodology establishes a new benchmark for SOTA performance in food recognition on the CNFOOD-241 dataset.

**Conclusions:**

We pioneer the integration of a residual learning framework within the VMamba model to concurrently harness both global and local state features. The code can be obtained on GitHub: https://github.com/ChiShengChen/ResVMamba.

## Introduction

Food plays a crucial role in human life, and with the rise of modern technology and significant changes in lifestyle and dietary habits, there has been an increasing emphasis on food computing [1]. Within this field, food recognition is a key area of research in computer vision and machine learning. Despite significant advancements, food image classification still faces several challenges, including low inter-class variation and high intra-class variation, variations in lighting and viewpoint, and occlusion in plated dishes. These challenges often lead to misclassification, limiting the real-world applicability of food recognition models. Deep learning has demonstrated remarkable adaptability across various domains, enabling breakthroughs in fields ranging from financial modeling, geomechanics [2–4], supply chain optimization [5] to biomedical engineering [6–9]. In food classification, Convolutional Neural Networks (CNNs) and Vision Transformers (ViTs) have achieved promising results, but they often struggle with feature extraction in fine-grained food categories [10]. To address these challenges, we propose an approach that integrates Deep Residual Learning and Selective State Space Models, leveraging the strengths of both techniques to enhance feature representation and improve classification accuracy. In industry, food image classification can be utilized for automating restaurant cooking processes, enabling self-checkout systems, and managing kitchen waste. Furthermore, food recognition is essential for various health-related applications, including nutritional analysis and dietary habit management.

In addition to the impacts of the photo-captured environment, noise in images, and image quality, the biggest challenge for food classification is low inter-class variation and high intra-class variation. The same food category can appear differently depending on cooking methods, seasonings, plating styles, and other preparation factors. While different food categories, even subtle differences in ingredients can result in visually similar but semantically different food types, such as shredded pork fried rice and shrimp fried rice. Addressing these issues requires techniques capturing fine-grained features to distinguish between food classes.

Fine-grained visual classification represents a formidable task within the field of computer vision, seeking to identify various subcategories within a broader category, such as the various species of birds [11], medical images [12], aircraft [13], pets [14], flowers [15], and natural images [16]. Food image recognition is also an important branch of Fine-Grained Visual Classification (FGVC) [17]. The pivotal point in the fine-grained classification is that in addition to learning global features, the model should be able to integrate local features to get global-local information mutually in order to achieve better recognition capabilities. Existing methods primarily focus on either developing model subnets for localizing discriminative features or improving feature learning strategies. However, achieving an optimal balance between local and global feature extraction remains a challenge.

In recent years, a new Sequence State Space (S4) model, through a Selection mechanism and computation with a Scan (S6), colloquially termed Mamba, has emerged as a promising alternative to Transformers due to their superior computational efficiency and capability to model long-range dependencies. The VMamba model [18], which incorporates the Mamba mechanism into image tasks (such as classification), currently establishes the state-of-the-art (SOTA) on the ImageNet dataset [19]. It retains the advantage of capturing both local and global information from input images as ViTs while also enhancing the model speed. However, there is still a lack of research on the application of VMamba to fine-grained datasets. Therefore, this study endeavors to employ VMamba on food images and introduces a model, ResVMamba, a novel model specifically designed for fine-grained datasets. Our approach, ResVMamba model, enhances the global-local feature integration capability of food image classification models by combining the efficiency of VMamba with residual learning mechanisms.

A well-defined dataset significantly influences the development of possible research topics and the feature-learning capabilities of models. In this study, we utilized the CNFOOD241 dataset [20]. CNFOOD241 is a Chinese food dataset created by expanding ChineseFoodNet [21], including correcting incorrect labels and increasing the number of images and food categories. In addition to model training, we provided a comparative analysis of CNFOOD241 and other food datasets, illustrating its suitability for research. Unlike other food databases, CNFOOD241 preserves the aspect ratio of images and standardizes the size to 600 × 600 pixels. This preprocessing step prevents image deformation during data augmentation, which could potentially lead to models learning incorrect semantic features. Furthermore, CNFOOD241 exhibits the relative imbalanced data distribution, making it a more challenging dataset for fine-grained food classification. By introducing ResVMamba, this study aims to advance FGVC in food recognition and provide a more efficient and accurate solution for real-world applications.

The contributions of this work are stated as follows:

- We provide comparative studies on the food dataset and clarify the research value of CNFOOD241. To enhance the rigor of our study, we have further partitioned the dataset into separate test and validation segments as a new fine-grained image classification benchmark.
- We first introduce the state space model into fine-grained image classification, and the proposed ResVMamba outperforms state-of-the-art approaches on the CNFOOD-241 dataset.

## Related work

### Food recognition datasets

In the burgeoning field of food computation, the proliferation of food datasets has marked a significant advancement, drawing widespread academic and practical interest. From the inception of datasets like ETH Food-101 [22], which introduced over one hundred thousand images of Western food varieties, to the expansive collections of ISIA Food-500 [23] and Food2K [24] encompassing nearly four hundred thousand and over a million images respectively, the evolution is notable. These datasets, predominantly sourced through web scraping, have been instrumental in advancing computational gastronomy and nutrition studies. However, they share a critical limitation: the lack of uniformity in the size distribution of images across different categories. This variance can lead to substantial discrepancies in some categories, where a few images might significantly exceed the average size of others, potentially skewing the dataset’s overall utility and introducing biases in the processing and classification results obtained after resizing images for analysis.

The issue of image size inconsistency poses challenges in maintaining the accuracy and reliability of computational models, especially those reliant on CNNs, ViTs and other image-processing architectures designed to extract detailed features from visual inputs. As depicted in **Table 1**, the disparity in image sizes may affect the performance of these models, leading to deviations in the extracted category-specific information and potentially impacting the overall effectiveness of the computational analysis.

**Table 1:** Image size statistics comparison of current open datasets of food recognition (pixels).

Dataset	Max. H	Max. H	Max. H	Min. H	Mean±Std. H x W
UEC Food100	800	71	800	80	358.25 ± 139.46 x 457.40 ± 180.58
UEC Food256	800	71	800	80	406.99 ± 117.16 x 492.98 ± 136.71
ETH Food-101	512	122	512	193	475.37 ± 65.31 x 495.79 ± 45.67
UPMC Food-101	2960	120	2000	120	459.13 ± 225.54 x 559.80 ± 268.67
Geo-Dish	-^*^	-^*^	-^*^	-^*^	-^*^
UNICT-FD889	240	240	320	320	240 ± 0.00 x 320 ± 0.00
Vireo Food-172	256	256	256	256	256 ± 0.00 x 256 ± 0.00
Food11	6144	207	9542	220	496.01 ± 180.38 x 531.55 ± 250.92
UNICT-FD1200	240	240	320	320	240 ± 0.00 x 320 ± 0.00
ChineseFoodNet	-^*^	-^*^	-^*^	-^*^	-^*^
Vegfru	6016	45	7360	48	309.73 ± 272.46 x 374.52 ± 338.21
Sushi-50	4752	232	6570	285	767.20 ± 584.50 x 955.80 ± 758.16
FoodX-251	2744	256	2733	256	287.48 ± 75.45 x 341.24 ± 86.28
ISIA Food-200	8868	70	10030	78	707.28 ± 561.74 x 830.91 ± 654.40
Taiwanese-Food-101	7360	61	6720	83	613.76 ± 372083 x 771.91 ± 455.90
ISIA Food-500	8868	100	10361	78	700.78 ± 566.41 x 822.08 ± 667.43
Food2K	1489	75	1717	97	381.26 ± 74.96 x 515.89 ± 102.72
**CNFOOD-241**	**600**	**600**	**600**	**600**	**600 ± 0.00 x 600 ± 0.00**

Max., Min., Std. are maximum, minimum and standard deviation respectively. H, W denotes the height and width of image respectively. The CNFOOD-241 dataset has the largest balenced image size in all the food image dataset we were available.

*The dataset download links provided on the official pages of Geo-Dish (http://isia.ict.ac.cn/dataset/Geolocation-food/) and ChineseFood-Net (https://goo.gl/kWNV8a) were inactive at the time of access.Thus, we were unable to obtain the datasets to analyze image properties.

In response to these challenges, our search for a more consistent and high-resolution dataset led us to CNFOOD-241. Among publicly available food datasets with uniform image sizes, such as UNICT-FD889 [25], Vireo Food-172 [26], and UNICT-FD1200 [27], CNFOOD-241 distinguishes itself by offering the highest resolution. This characteristic renders it an exceptional resource for conducting detailed image analyses within the food computation domain, facilitating more accurate and reliable studies in food recognition, nutritional analysis, and other related areas.

### Food image recognition

Early food recognition systems primarily used traditional machine learning algorithms. Researchers extract handcrafted features from images using methods such as color histograms [28], Scale-Invariant Feature Transform (SIFT), Histogram of Oriented Gradients (HOG) [29], Gabor textures [28], and Local Binary Pattern (LBP) [29]. These extracted features were then fed into classifiers such as SVM [30] for categorization. While achieving reasonable performance, these early methods relied heavily on manual feature engineering and were limited by image quality and variability in food appearances.

The emergence of deep learning revolutionized food identification research. Researchers began applying convolutional neural networks, such as AlexNet [31], ResNet-50 [32], EfficientNet, and Inception v3 [33], to food image data using transfer learning methods. This approach eliminated the need for manual feature extraction and allowed models to learn hierarchical visual representations from large-scale labeled datasets. Subsequent research enhanced food recognition capabilities using ensemble networks, multi-task learning, and other techniques. Notably, PRENet [24] emerged as a milestone in food recognition, integrating three different branches, each tailored for capturing different aspects of food images. By fusing features from low and high-level layers, PRENet achieved SOTA performance on CNN models.

Recently, ViT have gained popularity in food image analysis due to their ability to capture long-range dependencies. ViT models divide images into patches and represent them as sequence data, applying self-attention to capture relationships between patches. Ongoing research continues to integrate ViT with data augmentation, semi-supervised learning, multi-model fusion, and other techniques, pushing the boundaries of food understanding from images. Our research pioneers the application of the State Space Model to food recognition and aims to bring breakthroughs in this field.

### State space model on visual recognition

Recent research predominantly utilizes CNNs and ViTs for the task of classifying food categories. However, the capability of these models to detect features has not reached the SOTA in large-scale image classification challenges lately, being outperformed by a new generation of models known as Structured State Space for Sequence (S4) [34] modeling. The improvement of S4 models with a Selection mechanism and their execution using a Scan (S6) [35], informally known as Mamba, has been shown to outclass the Transformer architecture in handling long sequences. There are several Mamba models used on vision related task such as VMamba, Vision Mamba [18] have tried to use Mamba to do visual downstream tasks like image classification and object detection, but Vision Mamba more focus on inference speed and GPU-memory usage efficiency. U-Mamba [36], VM-UNet [37], and Mambamorph [38] have replaced convolutional blocks or downsample blocks to Mamba blocks applied on medical image segmentation tasks. However, it remains insufficient exploration of VMamba for fine-grained data and food recognition tasks, we therefore propose the VMamba-based model into these downstream tasks.

### Deep residual learning on space state model

Deep residual learning is a technique employed in training deep neural networks that offers several notable advantages introduced by ResNet [39]. Its primary advantage lies in addressing challenges such as vanishing gradients and exploding gradients, which commonly impede the training of deep networks. By introducing residual blocks and skip connections, deep residual learning facilitates the flow of gradients throughout the network, effectively mitigating the issue of vanishing gradients. Consequently, it enables the training of deeper neural networks without sacrificing performance. Deeper networks afford the extraction of more complex features, thereby enhancing the model’s representational capacity. Additionally, the training process in deep residual learning converges more efficiently due to expedited gradient propagation via skip connections, resulting in reduced training time and computational costs. However, to our best knowledge, there is lack of research that use residual learning on VMamba. Hence, we introduce the residual learning structure into VMamba-based model in this work.

## Methods

In this section, we first introduce the preliminary knowledge of VMamba, then propose the details of our ResVMamba structure.

### State space models

State Space Models (SSMs) are widely recognized as linear systems with time-invariant properties, mapping an input $x(t)\in \;R^{L}$ to an output $y(t)\in \;R^{L}$. These systems are mathematically formulated as linear ordinary differential equations (ODEs), as depicted in Equation (1), where the model’s parameters are denoted by $A\in \;C^{NxN}$, $B\in \;C^{NxN}$, for a system state of dimension *N*, and the direct link, D∈ CL. The state’s derivative and output signals are described by the following equations:


$$h^{\prime }(t=\;Ah\;(t)\;+\;Bx(t)$$

y(t= Ch (t) + Dx(t) (1)

### Discretization

When integrated into deep learning algorithms, State Space Models (SSMs), inherently continuous-time constructs, present substantial challenges. The discretization process is thus imperative.

The primary aim of discretization is to transmute the continuous ODE into a discrete function. This conversion is vital for aligning the model with the input data’s sample rate, thereby enabling computationally efficient operations [40]. Given the input x_k_ ∈ R^*L×D*^, which is a sampled vector from the signal sequence of length *L*, the ODE [41] (Eq. 1) can be discretized employing the zeroth-order hold approach:


$$h_{k}=\;A_{d}h_{k-1}\;+\;B_{d}x_{k}$$

$$y_{k}\;=\;C_{d}h_{k}\;+\;Dx_{k}\;$$ (2)

where $A_{d}=e^{A\Delta }$, $B_{d}={(e}^{A\Delta }-I)A^{-1}B$, and $C_{d}=C$, with $B,C\in \;{\mathbb{R}}^{D\times N}$ and $\Delta \in \;{\mathbb{R}}^{D}$. Following the practice, the approximation of B through first-order Taylor series is refined as:


$$B=\;(e^{A\Delta }-\;I)A^{-1}B\;\approx \;(\Delta A){\left(\Delta A\right)}^{-1}\Delta B\;=\;\Delta B$$ (3)

### 2D selective scan mechanism

The VMamba model introduces a novel Selective Scan Mechanism (S6), diverging from traditional Linear Time- Invariant (LTI) State Space Models (SSMs). This S6 mechanism, central to the VMamba framework, incorporates matrices $B\in \;{\mathbb{R}}^{B\times L\times N}$, $C\in \;{\mathbb{R}}^{B\times L\times D}$, and $\Delta \in \;{\mathbb{R}}^{B\times L\times D}$, extracted from the input data $x\in \;{\mathbb{R}}^{B\times L\times D}$, to imbue the system with contextual responsiveness and weight dynamism.

Furthermore, the Cross-Scan Module (CSM) is introduced to enhance spatial integration across the image. It unfolds image patches into sequences along rows and columns, and performs scanning across four directions, thereby enabling any pixel to integrate information from all others in different trajectories. These sequences are then reconfigured into a single image, culminating in a merged, information-rich new image.

### VMamba model

**Fig 1:**
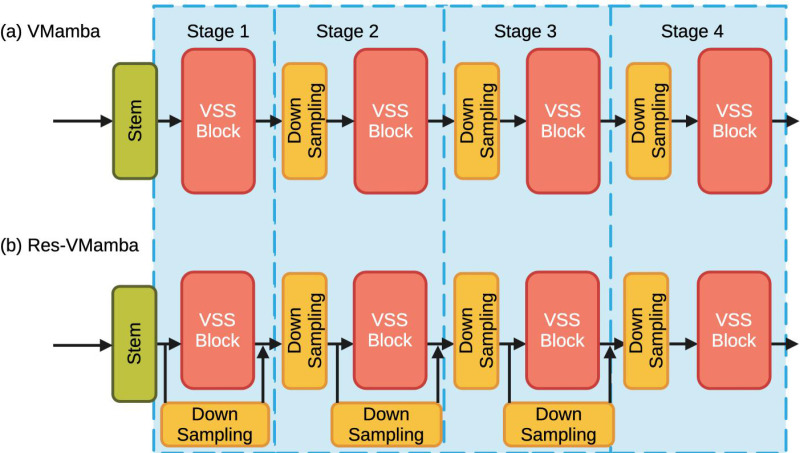
The comparison between VMamba and our proposed ResVMamba.

**Fig 2:**
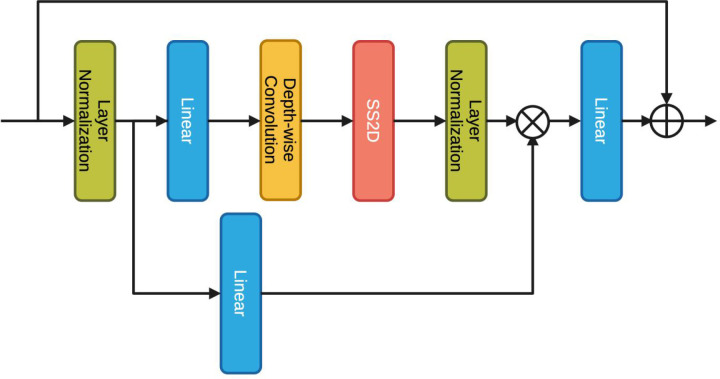
VSS Block.

The overall architecture of VMamba Model has been illustrated in previous literature [18]. The VMamba architecture, showcased in **Fig 1**, commences by partitioning the input image into patches through a stem module, emulating ViTs while maintaining the 2D structure and translating the patches into a 1D sequence. This approach yields a feature map with dimensions $\frac{H}{4}\;\times \;\frac{W}{4}\times \;C_{1}$. VMamba layers a series of VSS blocks like **Fig 2** atop this feature map to construct “Stage 1,” preserving its dimensions. Hierarchical structures in VMamba are established via down-sampling in “Stage 1” through a patch merging process. More VSS blocks are then integrated, reducing the output resolution to $\frac{H}{8}\;\times \;\frac{W}{8}$ for “Stage 2.” This down-sampling is reiterated to form “Stage 3” and “Stage 4,” with resolutions of $\frac{H}{16}\;\times \;\frac{W}{16}$ and $\frac{H}{32}\;\times \;\frac{W}{32}$, respectively. The resulting hierarchical design mirrors the multi-scale representation characteristic of renowned CNN models and some ViTs. Thus, VMamba’s architecture emerges as a comprehensive and versatile candidate for a variety of vision-related applications with analogous requirements.

### ResVMamba model

Inspired by ResNet, we have proposed a new type of VMamba model, ResVmamba, a Mamba with residual learning mechanism. ResVMamba architecture, as illustrated, is an advanced model configuration designed for efficient processing within the realm of computer vision. This architecture begins with a stem module that processes the input image, which is then followed by a series of VSS Blocks, arranged sequentially across four distinct stages.

Distinct from the original VMamba framework, the ResVMamba architecture not only employs the VMamba structure as its backbone but also integrates raw data directly into the feature map. In order to distinguish it from the residual structure in the VSS block, we called that global-residual mechanism. This integration is anticipated to facilitate the sharing of global image features in conjunction with the information processed through the VSS blocks. The intention behind this design is to harness both the localized details captured by individual VSS blocks and the overarching global features inherent in the unprocessed input, thereby enriching the model’s representational capacity and enhancing its performance on tasks requiring a comprehensive understanding of the visual data.

### Implementation details

**Fig 3:**
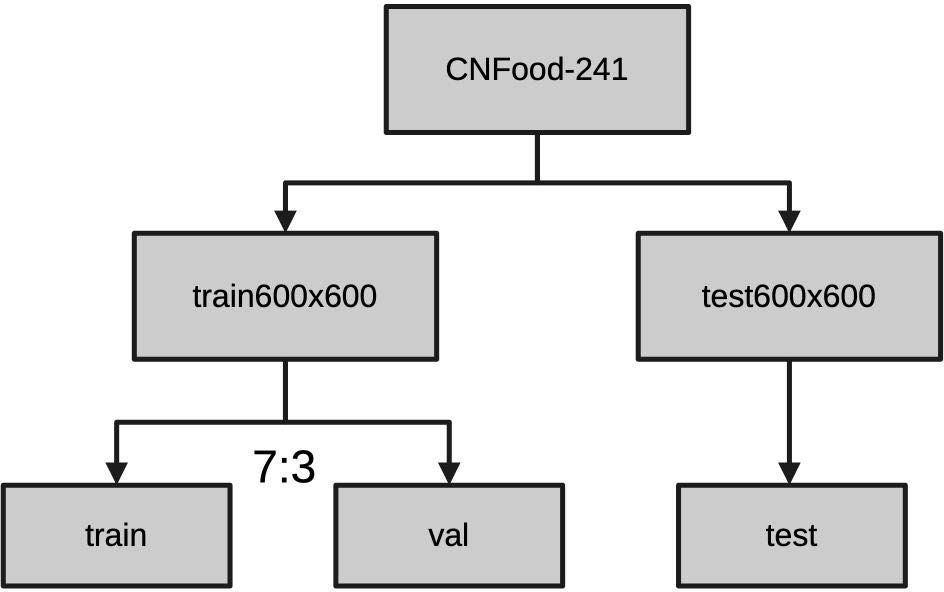
CNFOOD-241 data split flow.

In accordance with the protocol proposed in previous work [18], ResVMamba embarks on a comprehensive training regimen on CNFOOD-241, the backbone uses the VMamba-S, extending over 150 epochs with an initial warmup period covering the initial 20 epochs, and leverages a batch size of 128. The training schema integrates the AdamW optimizer, with the beta parameters set at (0.9, 0.999), and momentum fixed at 0.9. A cosine decay schedule modulates the learning rate, commencing with an initial learning rate of 1 × 10^-3^ and a weight decay parameter of 0.05. Augmenting the training are methodologies such as label smoothing at 0.1 and the implementation of an exponential moving average (EMA). Subsequent to these specified techniques, no further training strategies are deployed. The VMamba-S did transfer training with pretrained weight get from Liu et al. [18] on CNFOOD-241 with a batch size of 32, else training strategies is the same as default VMamba. The CMAL-Net are trained from ResNet-50 pretrained weight from pytorch based on its original setting on github, the others models are trained with pretrained weights on ImageNet-1K loaded from Huggingface by trim module with an initial learning rate of 1 × 10^−4^ and AdamW optimizer. Moreover, to establish CNFOOD-241 as a more equitable benchmark, we partitioned the dataset into training, validation, and test subsets at random. The training and validation sets were divided in a 7:3 ratio, respectively, through a randomized selection process. Finally, the training set contains 119,514 images and the validation set has 51,354 images in all 241 categories. The testing set is the original’val600x600’ folder in CNFOOD-241 dataset, which include 20,943 images as **Fig 3**. The train-validation split list can be available on the github: https://github.com/ChiShengChen/ResVMamba.

## Results

### Dataset

In this work, to fairly evaluate the models’ performance, we use the CNFOOD-241 as it possesses several notable characteristics as below:

### Balanced distribution on image sizes across categories

The CNFOOD-241 dataset possesses the largest (almost two hundred thousand images) uniform-sized (600 × 600) image collection among publicly available food datasets.

### Unbalanced number of images across categories on CNFOOD-241

In order to evaluate the imbalance in the number of images per category through the dataset, we calculate the normalized entropy. To normalize the entropy, we divide the entropy H by $\log_{2}(n)$, where n is the total number of categories. This normalized entropy, denoted as $H_{norm}$, is calculated as follows:


$$H_{norm}=\;\frac{H}{\log_{2}(n)}$$ (4)

Given the original definition of entropy:


$$H=-\sum_{i=1}^{n}p_{i}\log_{2}\left(p_{i}\right)$$ (5)

The normalized entropy $H_{norm}$ ranges from 0 to 1, where $H_{norm}$ = 1 indicates a perfectly balanced dataset, with each category having an equal share of the data. $H_{norm}$ = 0 indicates complete imbalance, where all instances belong to a single category. This normalization allows for an easier comparison of entropy values across datasets with different numbers of categories, providing a standardized measure of category balance. The entropy result is shown as **Table 2**, we observe that CNFOOD-241 exhibits a greater degree of class imbalance compared to other datasets, which consequently enhances the challenging nature of this dataset.

**Table 2:** comparison of entropy on current food recognition datasets.

Dataset	Category Entropy	Type	Public
UEC Food100	0.9758	Japanese	V
UEC Food256	0.9891	Japanese	V
ETH Food-101	1.0000	Western	V
UPMC Food-101	0.9999	Western	V
UNICT-FD889	0.9832	Misc.	V
Vireo Food-172	0.9876	Chinese	V
Food11	0.9565	Misc.	V
UNICT-FD1200	0.9883	Misc.	V
Vegfru	0.9759	Misc.	V
Sushi-50	0.9951	Japanese	V
FoodX-251	0.9974	Misc.	V
ISIA Food-200	0.9889	Misc.	V
Taiwanese-Food-101	1.0000	Chinese	V
ISIA Food-500	0.9880	Misc.	V
Food2K	0.9821	Misc.	V
CNFOOD-241	**0.9780**	Chinese	V

### Performance metrics

The performance of the models is evaluated by the top-k accuracy. Top-k accuracy is defined as the proportion of test samples for which the correct label is among the top k labels predicted by the model. Mathematically, it can be expressed as:


$$Top-k\;Accuracy\;=\;\frac{1}{N}\sum_{i=1}^{N}⊮\left(y^{\left(i\right)}\in P_{k}^{\left(i\right)}\right)$$ (6)

where:

- N is the total number of samples in the test set.
- $y^{\left(i\right)}$ is the true label for the *i*-th sample.
- $P_{k}^{\left(i\right)}$ is the set of top k predictions made by the model for the *i*-th sample.
- ⊮ is the indicator function, which is 1 if $y^{\left(i\right)}\in \;P_{k}^{\left(i\right)}$ (the true label is among the top k predictions) and 0 otherwise.

This metric is particularly useful for evaluating models on tasks where the goal is to provide a set of potential labels for each input and the exact rank within the top k is not critically important.

### Comparisons with State-of-the-Art (SOTA) methods

In the result we observe that VMamba-S with ImageNet-1k pretrained weight can reach the SOTA on CNFOOD-241, the ResVMamba surpass the VMamba-S and further improves the classification accuracy to 81.70%. (**Table 3**).

**Table 3:** Comparison of our approach (ResVmamba) to other baselines on CNFOOD-241 with our split method.

Model	Year	Use PW	Top-1 Test Acc.	Top-5 Test Acc.
VGG16	2014	Y	65.06	89.60
ViT-B	2020	Y	71.58	91.62
ResNet101	2015	Y	72.59	93.16
DenseNet121	2016	Y	74.77	94.29
InceptionV4	2016	Y	75.70	93.89
PRENet	2017	Y^*^	76.02	94.61
SEnet154	2017	Y	76.02	94.61
RepViT	2023	Y^†^	76.86	95.02
ConvNeXT-B	2022	Y	76.76	93.90
EfficientNet-B6	2019	Y	78.48	94.22
CMAL-Net	2023	Y	78.56	95.40
VMamba-S	2024	Y^‡^	80.58	96.71
**ResVMamba (Our model)**	**2024**	**Y** ^‡^	**81.70**	**96.83**

PW., Val., Acc. denotes pretrained weight, validation, accuracy, respectively.

*PRENet’s pretrained weight got from official github is trained on Food2K dataset.

† CMAL-Net used the ResNet-50 pretrained weight from pytorch, and other models’ pretrained weights got from timm module loaded from Huggingface trained on ImageNet-1K dataset.

‡ VMamba-S and ResVMamba pretrained weight got from official github is trained on ImageNet-1K dataset.

## Discussion

In the domain of computer vision, food recognition is categorically placed within the realm of FGVC, a field distinguished by its focus on distinguishing between closely related subcategories within a broader category. This area has seen the development and application of several state-of-the-art (SOTA) models, each contributing to advancements in dataset-specific performance. By comparing the CNFOOD-241 dataset with other food databases, we highlighted the characteristics of CNFOOD241 as a high-resolution, data-imbalanced, and therefore a challenging dataset. For the datasets of Food2K and ETH Food-101, PRENet achieves top-1 accuracies of 83.75% and 91.13%, respectively. However, its top-1 accuracy on CNFOOD-241 is only 76.2%, demonstrating the considerable difficulty of CNFOOD-241. The significant drop in PRENet’s performance (from 91.13% on ETH Food-101 to 76.2% on CNFOOD-241) highlights the dataset’s complexity. This challenge primarily arises from the low inter-class variation (e.g., visually similar dishes such as different styles of dumplings) and high intra-class variation (e.g., the same dish appearing in different lighting conditions, angles, and occlusions). Such characteristics make CNFOOD-241 a more challenging benchmark for FGVC tasks, as models need to develop stronger discriminative feature learning capabilities.

The unveiling of the CNFOOD-241 dataset marks a significant advancement in fulfilling the essential demand for high-quality, uniform datasets within the domain of food computation, facilitating novel pathways for research and innovation. Experimental evidence indicates that this dataset presents a considerable challenge. To address the challenge, we introduce ResVMamba, which is an enhanced version of the original VMamba model, incorporating a residual deep learning structure to improve its performance in processing complex food dataset. Residual deep learning mitigates the vanishing gradient problem by allowing gradients to flow through the network more effectively, hence enabling the model to learn better representations, especially in deep architectures [39].

VMamba models [18] based on the State Space Model are regarded as outperforming ViTs on large image datasets like ImageNet [19]. This model is designed to improve performance on intricate image classification tasks. In this study, we successfully integrated the residual deep learning structure into the VMamba model. Our data show that VMamba has superior performance compared to CMAL-Net in fine-grained food recognition on CNFOOD241, with a notable improvement of 2.02% in top-1 accuracy. Furthermore, results indicated that incorporating a residual architecture on VMamba (ResVMamba) can further enhance accuracy by 1.12%, validating the effectiveness of deep residual learning in FGVC. Therefore, ResVMamba is well-suited for handling high-resolution and data-imbalanced scenarios, making it ideal for real-world applications in food recognition.

The improvement observed in ResVMamba can be attributed to its hybrid design, which leverages state space models (SSMs) to efficiently capture long-range dependencies while retaining CNN-like locality. Compared to ViTs, which rely on computationally expensive self-attention mechanisms, SSMs process sequences linearly in time complexity, making them particularly suitable for high-resolution food images in CNFOOD-241. Additionally, the integration of residual learning into VMamba contributes to improved model convergence and feature extraction. Residual connections facilitate gradient propagation, mitigating the vanishing gradient issue commonly encountered in deep architectures. This enables ResVMamba to better capture discriminative fine-grained details, such as subtle texture and shape differences in food images.

From our results, the CMAL-Net [42] stands out as the secondary SOTA on CNFOOD241, having been constructed by integrating three expert modules with a CNN-based backbone. Each expert module processes feature maps from specific layers, delivering both a categorical prediction and an attention region. This attention region not only highlights areas of interest within the images but also serves as a means for data augmentation for the other expert modules, thereby enhancing the model’s overall accuracy and robustness. EfficientNet [43] demonstrate an accuracy of 78.48% on CNFOOD241, utilizing Network Architecture Search (NAS) and Compound Model Scaling to optimize performance. ConvNeXT [44], inspired by the Swin Transformer [45] architecture, reimagines CNNs to surpass the Swin Transformer’s performance on ImageNet, marking a significant achievement in model design.

The experimental results confirm that VMamba, when enhanced with residual learning, can outperform ViTs and CNNs in fine-grained classification tasks. The introduction of a residual structure allows the model to retain both local texture details and high-level semantic features, addressing the challenges of intra-class variations in food images. Additionally, compared to CNN-based methods like ResNet and EfficientNet, our model leverages the sequence modeling capabilities of state space models to capture long-range dependencies more effectively. These results suggest that state space models have significant potential beyond traditional sequence modeling applications and can be further explored in other fine-grained classification domains.

Our proposed model ResVMamba sets a new benchmark for state-of-the-art (SOTA) performance in food recognition tasks, demonstrating its effectiveness in complex food dataset on the CNFOOD-241 dataset. Deep residual learning enhances the model’s generalization capabilities by effectively fitting training data while mitigating the risk of overfitting. Future research should continue exploring the classification capabilities of the ResVMamba model on a larger scale. Beyond food classification, our findings suggest that state space models (SSMs) with residual learning can serve as a promising alternative to traditional CNNs and ViTs in fine-grained visual classification (FGVC) across various domains, including medical imaging, biological species identification, and industrial defect detection. Future research can explore the extension of ResVMamba to these domains, as well as investigate techniques such as adaptive residual scaling or multi-scale feature fusion to further enhance model robustness and generalization.

While CNFOOD-241 is a challenging dataset for fine-grained food classification, its geographic and cultural diversity remains limited. Additionally, although ResVMamba demonstrates strong performance in this domain, further studies are needed to assess its effectiveness across other fine-grained visual classification tasks. Moreover, the computational cost of ResVMamba is higher than that of traditional CNN-based models, which may impact its deployment in resource-constrained environments. Future work should focus on expanding dataset diversity and optimizing computational efficiency to enhance model generalizability and practical applicability.

## Conclusion

This study proposes the ResVMamba model, which integrates residual learning into the VMamba architecture for the first time in fine-grained food classification. Our comparisons with other food databases demonstrate that ResVMamba outperforms current SOTA models in fine-grained food classification with an accuracy of 81.70%. We demonstrate the potential of state space models in food image analysis. This research pioneers the integration of a residual learning framework within the VMamba model, enabling the effective utilization of both global and local feature states, which enhances its capability to tackle complex food recognition tasks. Future work will explore the application of ResVMamba to multi-modal food analysis and its integration into nutritional assessment systems.
